# Supplementary material for: Matrix mechanical plasticity regulates cancer cell migration through confining microenvironments
Source: Nat Commun. 2018 Oct 8;9:4144. doi: 10.1038/s41467-018-06641-z (PMC6175826; doi:10.1038/s41467-018-06641-z)
Supplement: Supplementary file 1 — Supplementary Information [file 41467_2018_6641_MOESM1_ESM.pdf]

Matrix mechanical plasticity regulates cancer cell migration through confining microenvironments

Katrina M. Wisdom[1], Kolade Adebowale[2], Julie Chang[3], Joanna Y. Lee[1], Sungmin Nam[1], Rajiv Desai[4], Ninna Struck Rossen[5], Marjan Rafat[5], Robert West[6], Louis Hodgson[7], and Ovijit Chaudhuri[1*]

[1]Department of Mechanical Engineering, Stanford University, Stanford, CA 94305, USA

[2]Department of Chemical Engineering, Stanford University, Stanford, CA 94305, USA

[3]Department of Bioengineering, Stanford University, Stanford, CA 94305, USA

[4]School of Engineering and Applied Sciences, Harvard University, Cambridge, MA 02138, USA

[5]Department of Radiation Oncology, Stanford University, Stanford, CA 94305, USA

[6]Department of Clinical Pathology, Stanford University, Stanford, CA 94305, USA

[7]Gruss-Lipper Biophotonics Center, Albert Einstein College of Medicine, Department of Anatomy and Structural Biology, Bronx, NY 10461, USA

*Correspondence to: chaudhuri@stanford.edu

Supplementary Information

Supplementary Figures

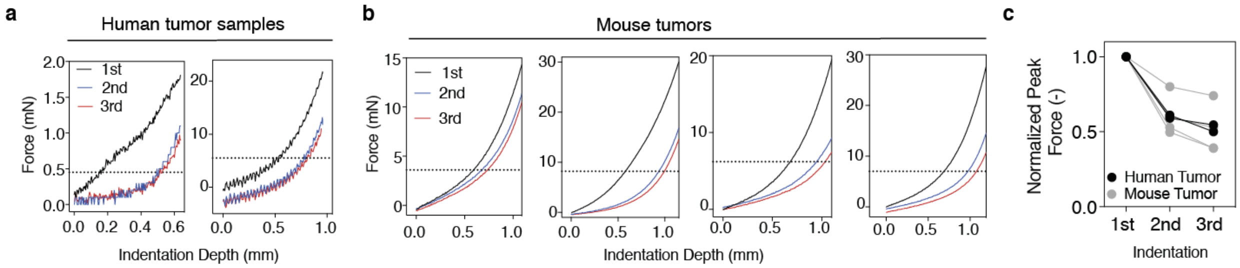

Supplementary Figure 1| Indentation test data of human and mouse tumor tissue show that these tissues exhibit mechanical plasticity. **a** and **b**, Force vs. indentation depth data for mechanical plasticity tests for all three indentations per sample. Dotted line indicates 25% of initial peak force. The rightward shift of indentation depth at this force magnitude determined the percentage of the indentation that was permanently retained, or indentation plasticity. **c**, Normalized peak forces of successive indentation cycles on human and mouse tumor tissue.

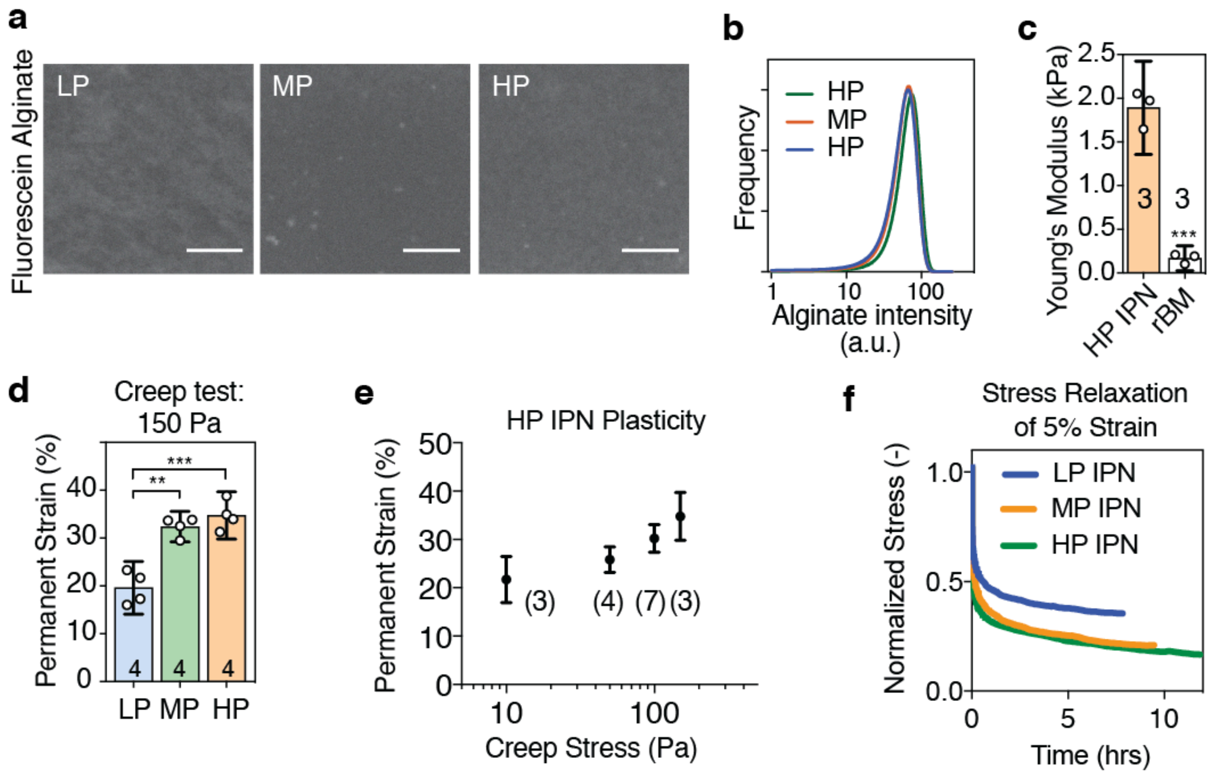

Supplementary Figure 2| IPN hydrogels are nanoporous, exhibit differential plasticity, and behave as viscoelastic solids. a, Images of fluorescent alginate in IPNs. Scale bar is 10 µm. **b**, Histograms of fluorescent alginate intensity per pixel taken from ten images. The single-peaked, narrow fluorescence intensity histograms indicate that the alginate is homogenously present at the resolution of images taken (~ 200 nm). These data also indicate that alginate component of the network is nanoporous. **c**, All IPNs (HP IPN shown here) have a significantly higher Young's modulus (i.e. initial stiffness) than that of rBM alone (*** $P < 0.001$, t-test). **d,** Creep (at 150 Pa) and recovery tests performed on IPNs. Differences in plasticity are significant as indicated (** $P < 0.01$, *** $P < 0.001$, ANOVA). **e**, Creep stress vs. permanent strain, or plasticity, for the HP IPN at 10, 50, 100, and 150Pa of creep. Creep period of 3600s and recovery period of 6400s were used for all studies. In **c-e**, plot shows mean and error bars indicate 95% confidence interval of the number of samples indicated. **f,** Stress relaxation test on all IPNs. As the IPNs relax over time spans longer than 7 hours, the normalized stress plateaus rather than diminishes to zero. As the

stress would be expected to go to zero in viscoelastic fluids, these data indicate that the IPNs behave like viscoelastic solids, and not fluids, on time scales relevant to cellular migration events.

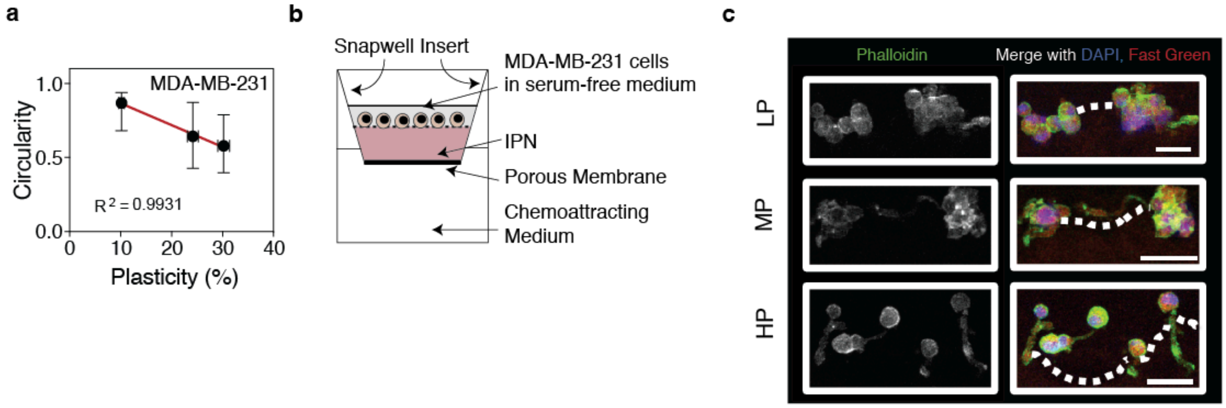

Supplementary Figure 3| MDA-MB-231 cancer cells exhibit differential morphologies when encapsulated in 3D, or plated on 2D, in invasion assays using IPN hydrogels. a, Circularity of MDA-MB-231 cells (median and interquartile range; n = 327, 337, and 347) versus plasticity (mean and S.E.M., n =3,3, and 3) of the IPNs in which they were encapsulated (R^2 = 0.9931 for trend line). **b**, Schematic of the experimental setup of the modified Boyden Chamber Invasion Assay. Snapwell insert membranes were coated with IPNs, and MDA-MB-231 cells were seeded on top of the IPN layer. Fresh invasion medium (50 ng/mL EGF) was deposited below the membrane every 1-2 days for 9 days. **c**, Confocal immunofluorescence imaging of stainings from cryosections of MDA-MB-231 cells seeded on IPNs of varying plasticity, fixed at day 9. Dotted line indicates the top of the IPN matrix, as determined by thresholding the fluorescence signal for the nonspecific matrix stain, FCF Fast Green, which fluoresces in the near infrared. Scale bar is 25 μm.

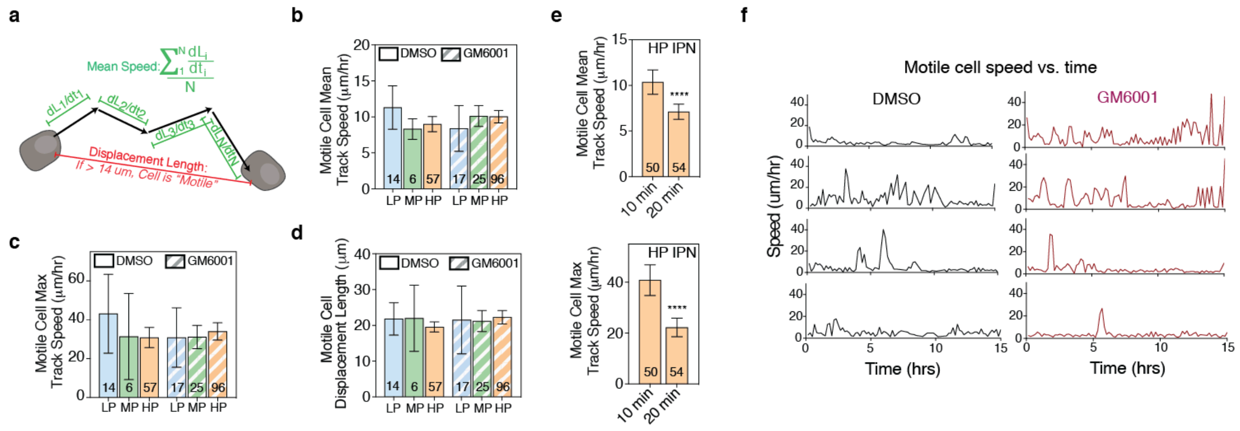

Supplementary Figure 4| Cancer cell motility characteristics in IPN hydrogels, with and without protease inhibitor. a, Criteria for identifying motile cells in IPNs, and definition of mean speed. **b**, Mean speeds, and **c**, maximum speeds for motile cells. **d**, Motile cell displacement lengths. For **b-d**, number of motile cells incorporated into these analyses are shown, pooled from R = 3-5 biological replicate experiments. Bars indicate means and error bars indicate 95% confidence intervals. No significant differences were observed (ANOVA). **e,** Example cell speed results, depending on the temporal resolution of the analysis (10 min. vs. 20 min. intervals) as indicated. As expected, the random cell motility within small channels is sensitive to temporal resolution of analysis (**** $P < 0.0001$, Student's t-test). **f,** Representative motile cell speed vs. time traces for cells in HP IPNs, for both vehicle alone and protease inhibitor conditions.

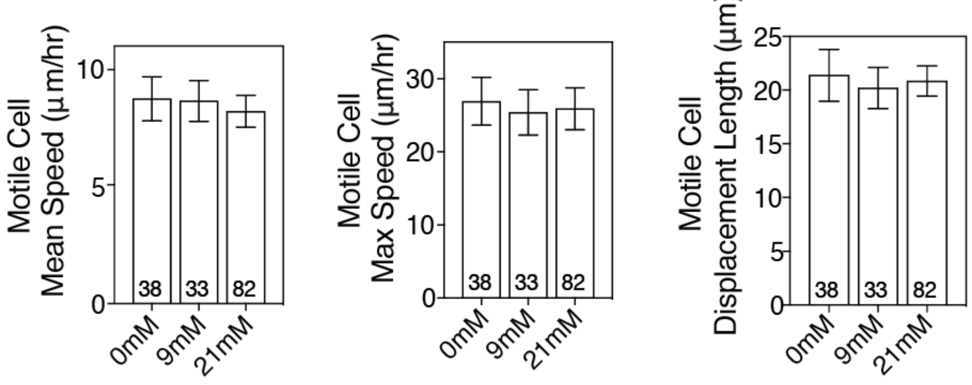

Supplementary Figure 5| Control migration assay studies in collagen-1 show that soluble calcium does not affect 3D cell motility. Motility characteristics for MDA-MB-231 cells encapsulated in collagen-1 hydrogels, with vehicle alone or calcium concentrations indicated, which are equivalent to those used to crosslink LP and HP IPNs, added to the medium. Motile cell mean, maximum speed, and displacement length do not show differences that are statistically significant (ANOVA). N = number of cells indicated from R = 2 biological replicate experiments.

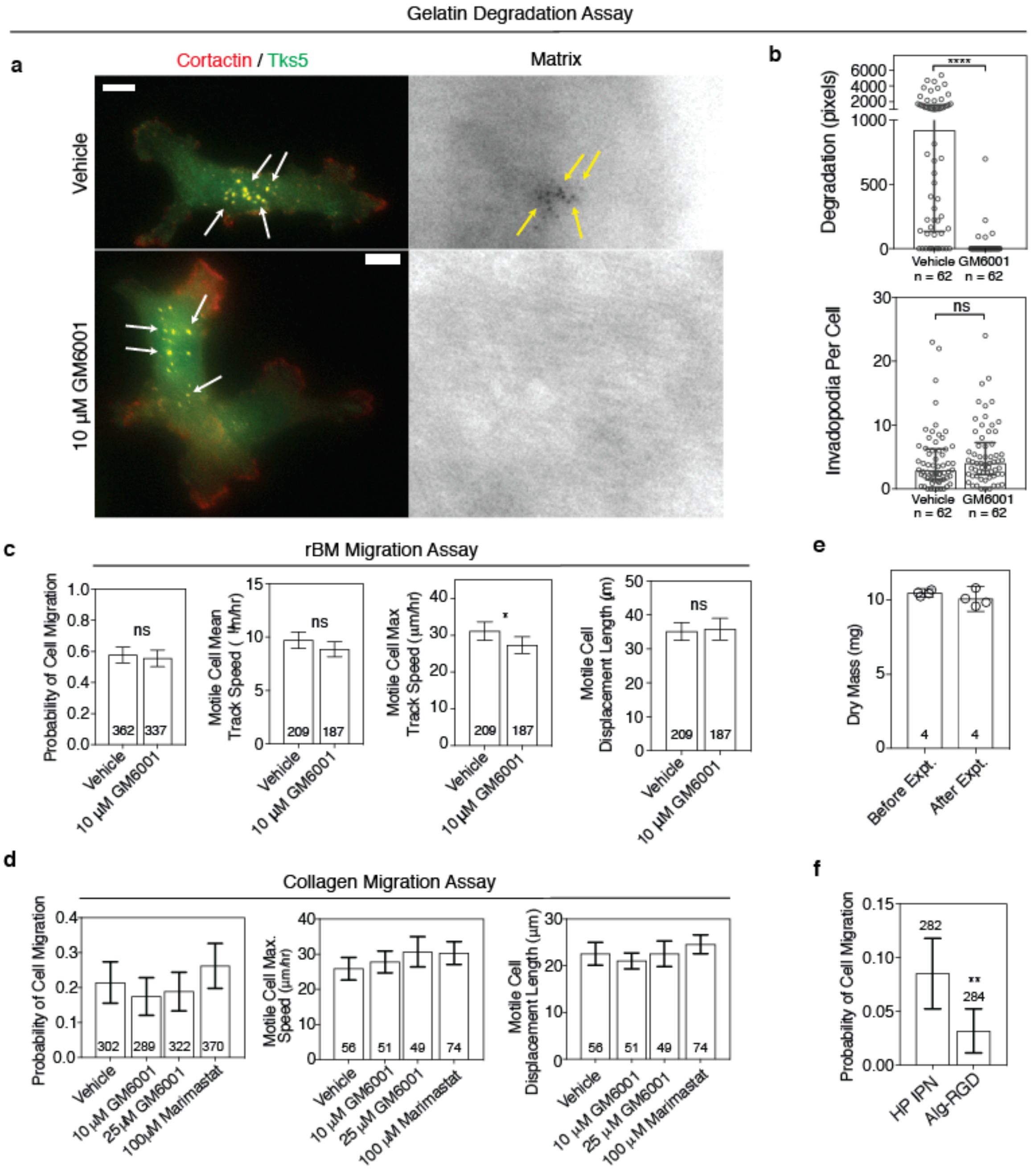

Supplementary Figure 6| Protease degradation, protease inhibitor efficacy, and migration control studies. a, MDA-MB-231 cells, with vehicle alone or protease inhibitor, were used in a classical gelatin degradation assay and stained for invadopodia markers cortactin (red) and TKS5 (green). White arrows (left) indicate colocalized cortactin and TKS5, and yellow arrows (right)

indicate regions of matrix degradation. Scale bar is 10 μm. **b,** Degradation area, in pixels, for the fields of view imaged, and invadopodia per cell, for vehicle alone and protease inhibitor conditions. Bars show medians and error bars indicate interquartile range of the data shown, which were taken from 3 independent biological replicates. Differences in degradation area are statistically significant (**** $P < 0.0001$, Kruskal-Wallis Test), confirming inhibitor efficacy. **c,** Motility characteristics of MDA-MB-231 cells encapsulated in 8 mg/mL rBM, with vehicle alone or protease inhibitor at the dosage indicated, added to the invasion medium. Data from 2 independent biological replicate experiments. The only significant difference observed was in motile cell maximum track speed, as indicated (* $P < 0.05$, t-test). **d**, 4 mg/mL collagen, with vehicle alone or protease inhibitor at the dosage indicated, added to the invasion medium, from 3 independent biological replicate experiments. No significant differences were observed (ANOVA). **e,** Alginate degradation control study. Panel displays dry mass of alginate hydrogel, for either gels frozen and lyophilized immediately after MDA-MB-231 encapsulation ("Before Expt.") or after an additional 2 days of culture ("After Expt."), similar to the time-lapse invasion assays. No significant differences were observed (t-test). **f**, Probability of cell migration for MDA-MB-231 cells encapsulated in HP IPN or 10 mg/mL RGD-conjugated alginate (i.e. without rBM the component present in the IPN). Data includes the number of cells indicated from 3 independent biological replicate experiments. For **c-f**, bars indicate mean and error bars indicate 95% confidence interval.

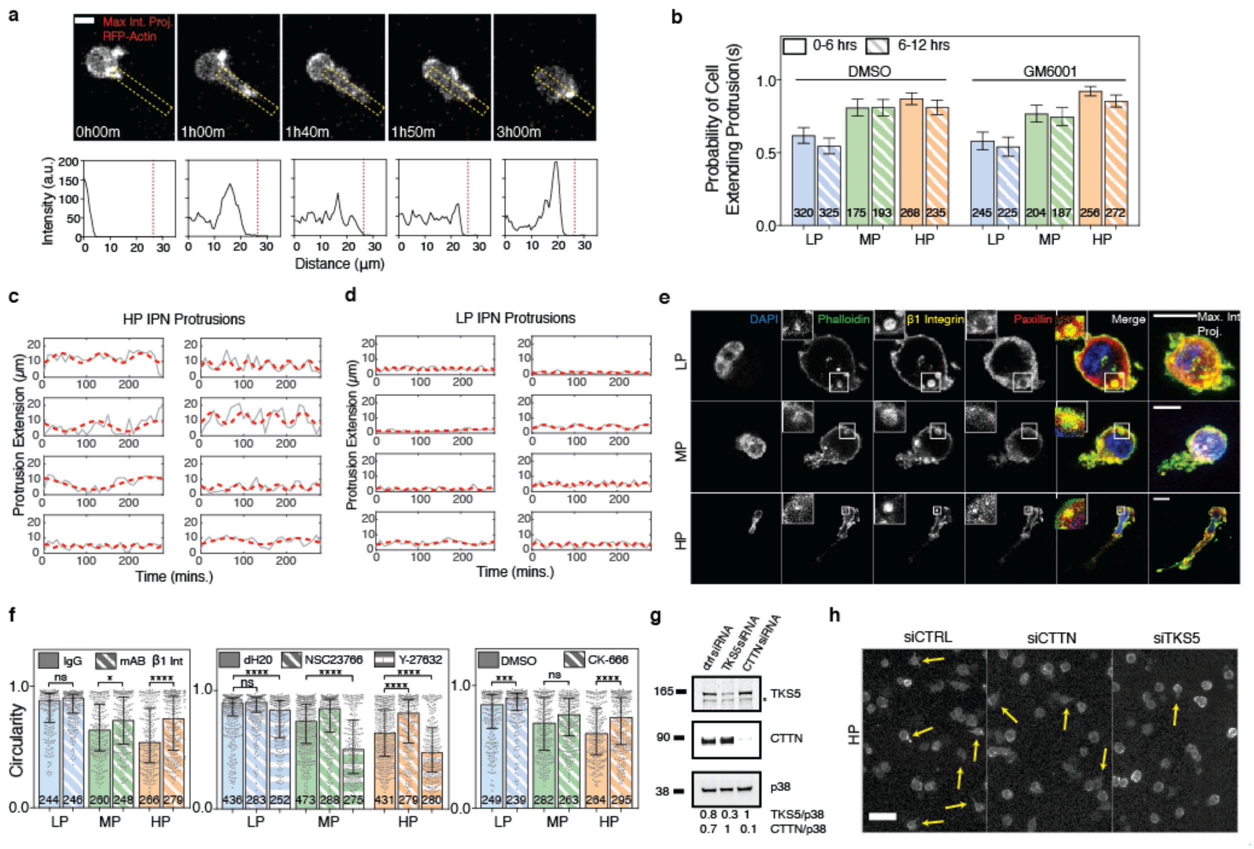

Supplementary Figure 7| Invasive protrusions in HP IPNs exhibit features and dependencies of classical invadopodia. a. Representative migrating MDA-MB-231 cell, transfected with RFP-LifeAct, with the last protrusion it extended prior to migrating. RFP-LifeAct signal intensity along the migration channel was measured at each time point as shown. Scale bar is 10 μm. **b**, Probability of MDA-MB-231 cells extending protrusions in all IPNs, vehicle alone and protease inhibitor conditions, assessed during the first and second parts of the imaging time window. **c** and **d,** Invadopodia extension distances over time were measured for 8 different cells in LP IPNs and HM IPNs. Sinusoidal fits to protrusion traces are shown with red dashed lines. **e**, Staining of DAPI (blue), actin (green), β1 integrin (yellow), and paxillin (red). Main panel scale bar is 10 μm, inset is 2x zoom. **f**, Quantification of MDA-MB-231 cell circularity in IPNs, with the indicated vehicle alone or inhibitor added to the media. Inhibitors used were: 1 μg/mL monoclonal β1 integrin

blocking antibody; 70 μM NSC23766 to inhibit Rac1 and 10 μM Y-27632 to inhibit ROCK; and 100 μM CK-666 to inhibit Arp 2/3. Data taken from R = 2 biological replicate experiments. Bars indicate median circularity of number of cells per condition as shown and error bars indicate interquartile range. The validity of comparing medians of pooled data sets was verified (Supplementary Tables 2 and 3). Significance among medians in cell circularity as shown (* $P < 0.05$, *** $P < 0.001$, **** $P < 0.0001$, Kruskal-Wallis). Inhibition of RhoA increased protrusivity, though this may be due to the known activation of Rac1 by RhoA inhibition[50]. **g**, Western blot analysis of MDA-MB-231 cells stably expressing RFP-LifeAct, transfected with cortactin siRNA (CTTN SMARTpool), TKS5 siRNA (SH3PXD2A SMARTpool), or control siRNA (ON-TARGETplus Non-Targeting Control Pool) for 72 hrs. Blots were stained for cortactin (CTTN), TKS5 (TKS5), and p38 (loading control). **h**, Representative maximum intensity projections of 3D stacks transfected cells as indicated. Yellow arrows indicate cellular protrusions. Scale bar = 50 μm.

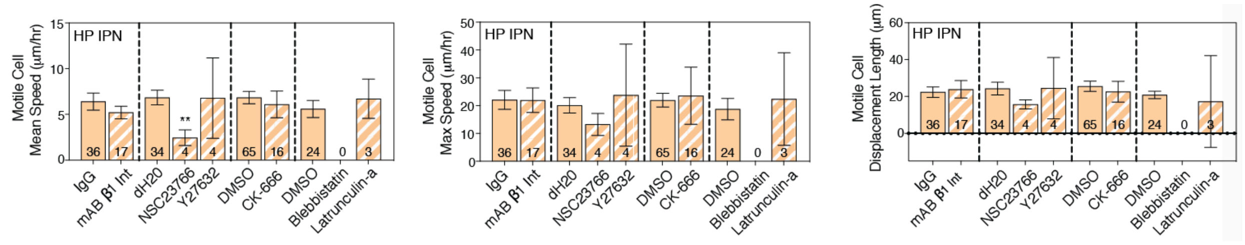

Supplementary Figure 8| Migratory cell speeds and displacements in HP IPNs, with the addition of inhibitors for key force-generation pathways. Quantification of motile cell mean speed, maximum speed, and displacement length with the addition of vehicle alone, antibody, or inhibitor. Bars indicate means and error bars indicate 95% confidence interval of the number of cells indicated, from R = 3 independent biological replicate experiments. One significant difference was found, in motile cell mean speed, with the vehicle alone vs. addition of Rac1 inhibitor NSC23766 (** $P < 0.01$, ANOVA).

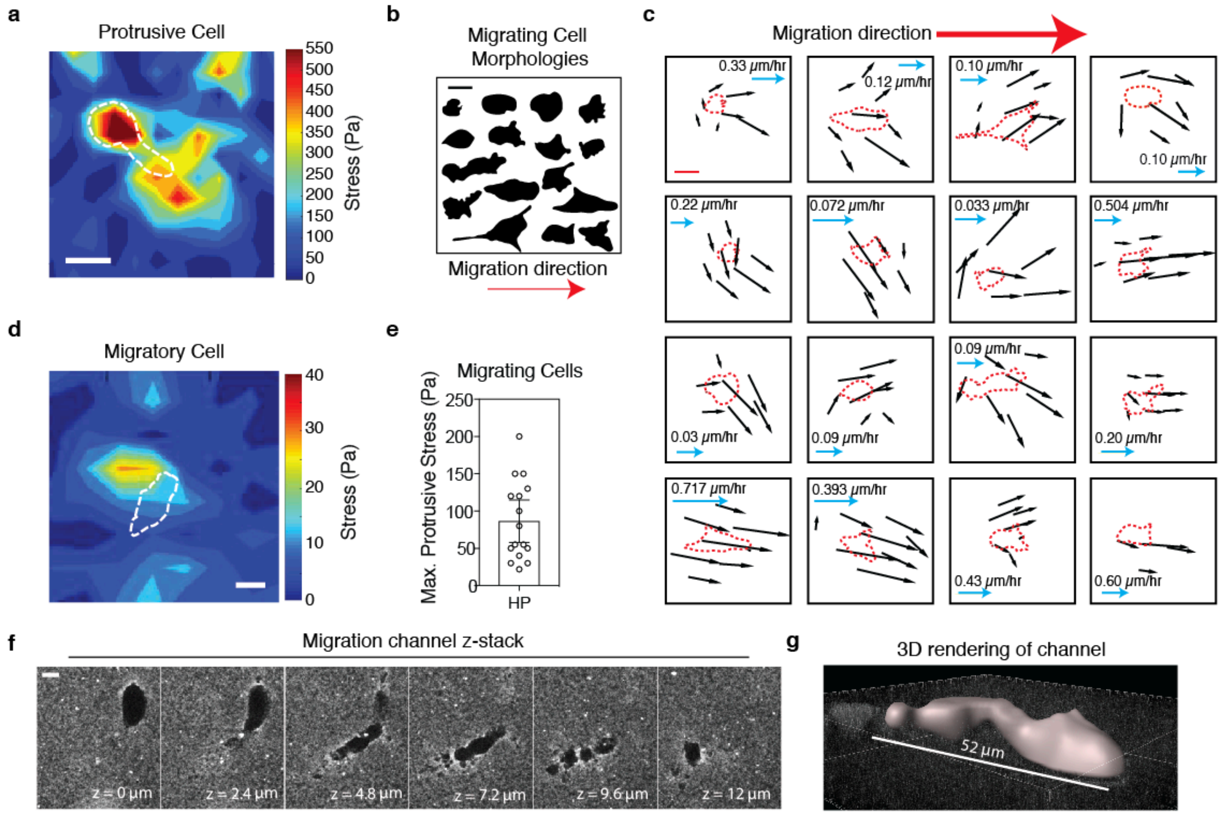

Supplementary Figure 9| Matrix displacement and stress analysis of protrusive and migratory cells. a, Stress heat map of a protrusive cell in HP IPN generated using a finite element analysis, based on matrix displacement maps. This analysis makes the assumption that the IPNs are elastic, thereby providing an upper bound on the actual mechanical stress. Scale bar is 20 μm. **b**, Shapes of the 16 migrating cells included in average matrix displacement map in Fig. 4c. **c**, 16 individual matrix displacement vector fields incorporated into Fig. 4c. Displacements were taken from within a cutoff radius from the corresponding cells, and were obtained by tracking fluorescent bead displacements around MDA-MB-231 cells migrating in HP IPNs. Cells and their vector maps were rotated so that all cells migrated to the right. Red scale bar, which denotes scaling of cells, is 20 μm. Matrix displacement speed scaling as indicated for each cell. **d**, Stress heat map of a migratory cell in HP IPN generated using finite element analysis. Scale bar is 20 μm. **e,**

Maximum protrusive stresses, generated using finite element analysis, exerted by migrating cells in HP IPNs. **f**, Images of fluorescent alginate in a HP IPN, taken from a z-stack of a migration channel that remained 3-4 hours after cell lysis and actin network depolymerization. Scale bar is 10 μm. **g**, Fluorescent alginate signal was inverted, and the empty channel was rendered in 3D. The end-to-end distance of the 3D rendered channel is 52 μm.

SUPPLEMENTARY TABLES

Description	Alginate Conc. (mg/mL)	Matrigel Conc. (mg/mL)	MW of Alginate (kDa)	Calcium Cross-linker Conc. (mM)	Initial elastic modulus (kPa, mean +/- s.d.)	Loss Tangent (unitless, mean +/- s.d.)	Plasticity (%, mean +/- s.d.)
Low Plasticity IPN	10	4.4	280	9	1.8 +/- 0.7	0.05 +/- 0.01	10 +/- 1
Medium Plasticity IPN	10	4.4	70	12	1.7 +/- 0.8	0.08 +/- 0.01	23 +/- 3
High Plasticity IPN	10	4.4	35	24	1.8 +/- 0.6	0.10 +/- 0.02	30 +/- 2

Supplementary Table 1| List of example hydrogel formulations used in study. IPN formulations were periodically re-tested and re-tuned to account for variation in rBM batches.

Data	Figure	Test For (Test Used)	Assumptions on normality, variance, etc.	**Test Details** (α = 0.05 unless otherwise indicated)
**Mechanical Testing**				
**Loss Tangent**	1g	1. Compare means (ANOVA with Sidak's Multiple Comparison Test).	Normality and equal variances	P < 0.0001, F(2,33) = 48.97
		2. Test for trend (Spearman's Rank Correlation)	Normality and linear trend	P (two-tailed) < 0.0001<br>R = 0.8469
**Plasticity**	1j	1. Compare means (ANOVA with Sidak's Multiple Comparison Test).	Normality and equal variances	P < 0.0001, F(2,13) = 58.67
		2. Test for trend (Spearman's Rank Correlation)	Normality and linear trend	P (two-tailed) < 0.0001<br>R = 0.8657
	1k	1. Compare means (ANOVA with Sidak's Multiple Comparison Test).	Normality and equal variances	P < 0.0001, F (3,8) = 186.6
	Supp. 2d	Compare means (ANOVA with Sidak's Multiple Comparison Test).	Normality and equal variances	P < 0.0001, F(2,9) = 31.17
**Young's Modulus**	Supp. 2c	Compare means (Student's t-test)	Normality and equal variances	P (two-tailed) = 0.0002
**Invasive Morphology**				
By Cell Line	2c	Compare medians (Mann-Whitney U-Tests)	Not normal and unequal variances	P (two-tailed) < 0.0001
Vehicle Alone vs. Protease Inhibitor	2d	1.Compare distributions within biological replicates to validate pooling data (Kolmogorov-Smirnov Tests)	Not normal and unequal variances	α = 0.01, two-tailed<br>See Extended Data Table 2.<br>*If null hypothesis not rejected, data sets drawn from same distribution, and pooled medians can be compared*
		2. Compare medians (Mann-Whitney U-Test)	Not normal and unequal variances	P (two-tailed) < 0.0001
Vehicle Alone vs. Other Inhibitors	Supp. 7f	1.Compare distributions within biological replicates to validate pooling data (Kolmogorov-Smirnov Tests)	Not normal and unequal variances	α = 0.01, two tailed<br>See Extended Data Table 2.<br>*If null hypothesis not rejected, data sets drawn from same distribution, and pooled medians can be compared*
		2. Compare medians (Kruskal Wallis with Dunn's Multiple Comparison Test)	Not normal and unequal variances	*Integrin:* P < 0.0001,<br>K-W Statistic = 215;<br>*Rac/Rock:* P < 0.0001,<br>K-W Statistic = 556.8;<br>Arp2/3: P < 0.0001,<br>K-W Statistic = 152.9
**Cell Motility**				
All IPNs, with and without protease inhibitor	2g	1. Compare number of motile cells vs. non-motile cells (Fisher's Exact Tests, corrected for multiple tests)	Binary outcome	*DMSO:* P (two-tailed) < 0.0001<br>*GM6001:* P(two-tailed) < 0.0001
		2. Test for trend (Chi-square test for trend)	Binary outcome	*GM6001*: P < 0.00001;<br>Chi-sq. stat, df = (52.23,1)
HP IPN + vehicle or force-generating pathway inhibition	4a	Compare number of motile cells vs. non-motile cells (Fisher's Exact Tests, corrected for multiple tests)	Binary outcome	*Lat-a:* P (two-tailed) = 0.0044<br>*Others*: P (two-tailed) < 0.0001
HP IPN + vehicle or force-generating pathway inhibition	Supp. 8	Compare mean and max speeds and displacement lengths (ANOVA)	Normality and equal variances	*NSC23766 Mean Speed*: P = 0.003
Migration speed, by temporal resolution	Supp. 4e	Compare mean speeds of motile cells (Student's t-test)	Normality and equal variances	*Both:* P (two-tailed) < 0.0001
Migration in rBM	Supp. 6c	Compare mean and max speeds and displacement lengths (t-tests)	Normality and equal variance	*Max Speed:* P(two-tailed) = 0.027
Migration probability, RGD-alginate	Supp. 6f	Compare number of motile cells vs. non-motile cells (Fisher's Exact Test)	Binary outcome	P = 0.007
**Probability of Cell Protrusivity**				
All IPNs	3b	1. Compare number of protrusive cells vs. non-protrusive cells (Fisher's Exact Tests)	Binary outcome	*DMSO:* P (two-tailed) < 0.0001<br>*GM6001:* P(two-tailed) < 0.0001
		2. Test for trend (Chi-square test for trend)	Binary outcome	*DMSO:* P < 0.0001;<br>Chi-sq. stat, df = (50.99,1)<br>*GM6001:* P< 0.0001;<br>Chi-sq. stat, df = (81.34,1)
HP IPN: siRNA control, siCTTN, siTks5	3i	Compare number of protrusive cells vs. non-protrusive cells (Fisher's Exact Tests, corrected for multiple tests)	Binary outcome	*siCTTN:* P (two-tailed) = 0.0066<br>*siTks5:* P(two-tailed) < 0.0001
**Other**				
**Matrix Displacements**	4e	Compare mean matrix displacements to vehicle alone controls (ANOVA)	Normality and equal variances	*Lat-a:* P (two-tailed) < 0.002<br>*Bleb:* P(two-tailed) < 0.0001
**Gelatin Degradation Assay**	Supp. 6b	Compare median degradation areas (Mann-Whitney U-Test)	Not normal and unequal variance	P(two-tailed) < 0.0001

Supplementary Table 2| Additional information on statistical tests performed.

Sample	K-S Test Pass? *(1=Yes, 0=No)*	P-Value
LP_DMSO	1	0.467
LP_GM6001	1	0.544
MP_DMSO	1	0.146
MP_GM6001	1	0.127
HP_DMSO	1	0.312
HP_GM6001	1	0.947
LP_IgG	1	0.784
LP_mAB_β1Int	1	0.774
MP_IgG	1	0.748
MP_mAB_β1Int	1	0.04
HP_IgG	1	0.013
HP_mAB_β1Int	1	0.634
LP_dH20	1	0.682
LP_NSC23766	1	0.032
LP_Y27632	1	0.025
MP_dH20	1	0.988
MP_NSC23766	0	3.10E-08
MP_Y27632	1	0.138
HP_dH20	1	0.01
HP_NSC23766	1	0.016
HP_Y27632	1	0.027
LP_DMSO2	1	0.034
LP_CK666	1	0.26
MP_DMSO2	1	0.235
MP_CK666	1	0.292
HP_DMSO2	1	0.075
HP_CK666	1	0.956

Supplementary Table 3| Validity of statistical comparisons of medians using pooled biological replicates. Circularity distributions for all MDA-MB-231 invasive morphology experiments were compared between replicates using a Kolmogorov-Smirnov test, with $\alpha = 0.01$. Statistical comparisons (i.e. Mann-Whitney or Kruskal-Wallis) were not performed between or among medians of pooled data if the K-S test rejected the null hypothesis that the two replicate data sets were drawn from the same distribution.
