# Supplementary material for: Matrix mechanical plasticity regulates cancer cell migration through confining microenvironments
Source: Nat Commun. 2018 Oct 8;9:4144. doi: 10.1038/s41467-018-06641-z (PMC6175826; doi:10.1038/s41467-018-06641-z)
Supplement: Supplementary file 21 — Description of Additional Supplementary Files [file 41467_2018_6641_MOESM21_ESM.docx]

**Title:** Supplementary Movie 1| Example cell in HP IPN, migrating.
**Description:** 3D reconstruction of confocal fluorescence timelapse stack (spanning 40 µm) of a cell (RFP-LifeAct MDA-MB-231) migrating in HP IPN, with vehicle alone added. Left panel is actin signal alone, and right panel is actin signal merged with bright field. Frames are taken every 10 minutes for over 14 hours.

**Title:** Supplementary Movie 2| Tracked cells in LP IPN.
**Description:** Cell centroid tracking of cells in a confocal fluorescence timelapse stack (spanning 40 µm) of RFP-LifeAct MDA-MB-231 cells in LP IPN, with vehicle alone added. Frames are taken every 10 minutes for over 14 hours.

**Title:** Supplementary Movie 3| Tracked cells in HP IPN.
**Description:** Cell centroid tracking of cells in a confocal fluorescence timelapse stack (spanning 40 µm) of RFP-LifeAct MDA-MB-231 cells in HP IPN, with vehicle alone added. Frames are taken every 10 minutes for over 14 hours.

**Title:** Supplementary Movie 4| Example cell migrating in HP IPN with protease inhibitor.
**Description:** 3D reconstruction of confocal fluorescence time-lapse stack (spanning 40 µm) of a cell (RFP-LifeAct MDA-MB-231) migrating in HP IPN, with 10 µM GM6001 added. Left panel is actin signal alone, and right panel is actin signal merged with bright field. Frames are taken every 10 minutes for over 14 hours.

**Title:** Supplementary Movie 5| Tracked cells in LP IPN with protease inhibitor.
**Description:** Cell centroid tracking of cells from a confocal fluorescence time-lapse stack (spanning 40 µm) of RFP-LifeAct MDA-MB-231 cells in LP IPN with 10 µM GM6001. Frames are taken every 10 minutes for over 14 hours.

**Title:** Supplementary Movie 6| Tracked cells in HP IPN with protease inhibitor.
**Description:** Cell centroid tracking of 3D rendered cells from a confocal fluorescence time-lapse stack (spanning 40 µm) of RFP-LifeAct MDA-MB-231 cells in HP IPN with 10 µM GM6001. Frames are taken every 10 minutes for over 14 hours.

**Title:** Supplementary Movie 7| Tracked cells in collagen-1 with vehicle alone.
**Description:** Cell centroid tracking of 3D rendered cells from a confocal fluorescence time-lapse stack (spanning 40 µm) of RFP-LifeAct MDA-MB-231 cells in 4 mg/mL collagen-1 with vehicle alone (DMSO). Frames are taken every 20 minutes for over 18 hours.

**Title:** Supplementary Movie 8| Tracked cells in collagen-1 with protease inhibitor GM6001.
**Description:** Cell centroid tracking of 3D rendered cells from a confocal fluorescence time-lapse stack (spanning 40 µm) of RFP-LifeAct MDA-MB-231 cells in 4 mg/mL collagen-1 with 10 µM GM6001. Frames are taken every 20 minutes for over 18 hours.

**Title:** Supplementary Movie 9| Tracked cells in collagen-1 with protease inhibitor Marimastat.
 **Description:** Cell centroid tracking of 3D rendered cells from a confocal fluorescence time-lapse stack (spanning 40 µm) of RFP-LifeAct MDAMB-231 cells in 4 mg/mL collagen-1 with 100 µM Marimastat. Frames are taken every 20 minutes for over 18 hours.

**Title:** Supplementary Movie 10| Actin dynamics of cells in LP IPN.
**Description:** Maximum intensity projection (MIP) of a confocal fluorescence time-lapse stack, with RFP-LifeAct raw data alone (left) and then pseudocolored and merged with bright field (right). Frames are taken every 5 minutes for over 1 hour. Scale bar is 10 µm.

**Title:** Supplementary Movie 11| Actin dynamics of cells in HP IPN.
**Description:** Maximum intensity projection (MIP) of a confocal fluorescence time-lapse stack, with RFP-LifeAct raw data alone (left) and then pseudocolored and merged with bright field (right). Frames are taken every 5 minutes for over 3 hours. Scale bar is 10 µm.

**Title:** Supplementary Movie 12| Close-up of tracked cells in HP IPN control.
**Description:** Cell centroid tracking of 3D rendered cells from a confocal fluorescence time-lapse stack (spanning 40 µm) of RFP-LifeAct MDA-MB-231 cells in HP IPN with vehicle alone. Frames are taken every 20 minutes for 15 hours.

**Title:** Supplementary Movie 13| Close-up of tracked cells in HP IPN with Rac1 inhibitor.
**Description:** Cell centroid tracking of 3D rendered cells from a confocal fluorescence time-lapse stack (spanning 40 µm) of RFP-LifeAct MDA-MB-231 cells in HP IPN with Rac1 inhibitor NSC23766. Frames are taken every 20 minutes for 11 hours.

**Title:** Supplementary Movie 14| Close-up of tracked cells in HP IPN with ROCK inhibitor.
**Description:** Cell centroid tracking of 3D rendered cells from a confocal fluorescence time-lapse stack (spanning 40 µm) of RFP-LifeAct MDA-MB-231 cells in HP IPN with ROCK inhibitor Y-27632. Frames are taken every 20 minutes for 11 hours.

**Title:** Supplementary Movie 14| Close-up of tracked cells in HP IPN with ROCK inhibitor.
**Description:** Cell centroid tracking of 3D rendered cells from a confocal fluorescence time-lapse stack (spanning 40 µm) of RFP-LifeAct MDA-MB-231 cells in HP IPN with ROCK inhibitor Y-27632. Frames are taken every 20 minutes for 11 hours.

**Title:** Supplementary Movie 15| Close-up of tracked cells in HP IPN with Arp 2/3 inhibitor.
**Description:** Cell centroid tracking of 3D rendered cells from a confocal fluorescence time-lapse stack (spanning 40 µm) of RFP-LifeAct MDA-MB-231 cells in HP IPN with Arp 2/3 inhibitor CK-666. Frames are taken every 20 minutes for 15 hours.

**Title:** Supplementary Movie 16| Close-up of tracked cells in HP IPN with Myosin-II inhibitor.
**Description:** Cell centroid tracking of 3D rendered cells from a confocal fluorescence time-lapse stack (spanning 40 µm) of RFP-LifeAct MDA-MB-231 cells in HP IPN with Myosin II inhibitor Blebbistatin. Frames are taken every 20 minutes for 16 hours.

**Title:** Supplementary Movie 17| Close-up of tracked cells in HP IPN with F-actin inhibitor.
**Description:** Cell centroid tracking of 3D rendered cells from a confocal fluorescence time-lapse stack (spanning 40 µm) of RFP-LifeAct MDA-MB-231 cells in HP IPN with F-actin inhibitor Latrunculin-a. Frames are taken every 20 minutes for 16 hours.

**Title:** Supplementary Movie 18| Cells in HP IPN with beads.
 **Description:** Showing the opening of a channel due to cell migration. 3D rendering of a confocal fluorescence time-lapse stack, with RFP-LifeAct (red) merged with embedded fluorescent beads (white). Frames are taken every 10 minutes for 12 hours. Part 1 shows XY view of a migrating cell (blue arrow), in relation to an XZ cross sectional plane (yellow, ~ 3 µm thick) intersecting the cell migration path. In Part 2, the perspective has been rotated to highlight the intensity in this cross sectional XZ plane (yellow) during the same migration event.
